# Effects of arbuscular mycorrhizal fungi on growth and nitrogen uptake of *Chrysanthemum morifolium* under salt stress

**DOI:** 10.1371/journal.pone.0196408

**Published:** 2018-04-26

**Authors:** Yanhong Wang, Minqiang Wang, Yan Li, Aiping Wu, Juying Huang

**Affiliations:** 1 State Key Laboratory of Subtropical Silviculture, Zhejiang A&F University, Hangzhou, Zhejiang, China; 2 Hunan Provincial Key Laboratory of Rural Ecosystem Health in Dongting Lake Area, Hunan Agricultural University, Changsha, Hunan, China; 3 Institute of Environmental Engineering, Ningxia University, Yinchuan, Ningxia, China; RIKEN Center for Sustainable Resource Science, JAPAN

## Abstract

Soil salinity is a common and serious environmental problem worldwide. Arbuscular mycorrhizal fungi (AMF) are considered as bio-ameliorators of soil salinity tolerance in plants. However, few studies have addressed the possible benefits of AMF inoculation for medicinal plants under saline conditions. In this study, we examined the effects of colonization with two AMF, *Funneliformis mosseae* and *Diversispora versiformis*, alone and in combination, on the growth and nutrient uptake of the medicinal plant *Chrysanthemum morifolium* (Hangbaiju) in a greenhouse salt stress experiment. After 6 weeks of a non-saline pretreatment, Hangbaiju plants with and without AMF were grown for five months under salinity levels that were achieved using 0, 50 and 200 mM NaCl. Root length, shoot and root dry weight, total dry weight, and root N concentration were higher in the mycorrhizal plants than in the non-mycorrhizal plants under conditions of moderate salinity, especially with *D*. *versiformis* colonization. As salinity increased, mycorrhizal colonization and mycorrhizal dependence decreased. The enhancement of root N uptake is probably the main mechanism underlying salt tolerance in mycorrhizal plants. These results suggest that the symbiotic associations between the fungus *D*. *versiformis* and *C*. *morifolium* plants may be useful in biotechnological practice.

## Introduction

Salinity is a serious environmental problem, and over 800 million hectares of the land surface worldwide are affected by excessive salt [1,2]. Saline soil in China covers approximately 34.6 million hectares, mainly distributed in northern China and the areas along the Changjiang River [3]. In addition to natural causes such as high salinity precipitation and weathering of native rocks, irrigation with poor-quality water, land clearing, low levels of precipitation, high temperatures and over-exploitation of available water resources have also exacerbated the increasing salinity levels in soils in many parts of the world [4–7]. Even worse, increasing salinization of arable land will result in the loss of 30% of arable land within the next 25 years and of up to 50% of arable land by 2050 [8–10]. High salinity levels can cause both hyperionic and hyperosmotic stress and result in decreased plants growth, lower nutrient uptake levels, and even death [5–7]. To address saline soils and minimize commercial plant loss, many approaches are employed to combat salt stress; of these approaches, the application of arbuscular mycorrhizal fungi (AMF) in saline soils has been considered as a bio-ameliorator in plants [7,10–12].

AMF are a normal and universal component of the rhizosphere microflora, and nearly 70~90% of land plant species in all terrestrial ecosystems can become colonized by these fungi [13–16]. AMF occur naturally in saline environments in association with native species, including halophytes, hydrophytes and xerophytes [7,13,17]. Many studies have indicated that AMF can improve plant growth and nutrient uptake under salt-stressed conditions [5,7,10,18–21]. Some studies have suggested that enhancing the uptake of nutrients, especially phosphorus (P), is the most important mechanism to address salinity stress tolerance in AMF plants [7,10], whereas other studies have reported reduced uptake of P by AMF-inoculated plants grown under saline conditions [5,22,23]. In addition, not all AMF improve plant growth in saline soils equally well [4,7,10,11]. For example, some researchers reported that *Glomus fasciculatus* appeared to be the fungus that was most efficient at reducing the negative effects of salinity [10,22], while others suggested that *G*. *mosseae* was a better option to reduce salt stress [7,24]. These inconsistent effects of AMF under salt stress could result from either the salt tolerance of the host species or the fungal species [7,22,25]. Furthermore, most studies on the interactions of mycorrhiza and salinity have been conducted with crop plants compared with few studies using medicinal plants [7,10,16].

Hangbaiju (*Chrysanthemum morifolium* (Ramat.) Tzvel), one cultivar of the genus *Dendranthema*, is a perennial herbaceous medicinal plant, and its economic importance in China has been attributed to the fact that the world’s largest producers and exporters of its flowers are in Zhejiang Province [26,27]. At the industrial scale, Hangbaiju is mainly distributed in Tongxiang City, Zhejiang Province; Sheyang City, Jiangsu province; Ma City, Hubei Province; and Xiushui City, Jiangxi Province, China, among which Tongxiang City is its geographical origin [28,29]. In addition to the unique taste of its flower as a tea and the young plants as a food, Hangbaiju plants contain biologically active compounds such as phenolics, mainly flavonoids, which contribute to disease prevention and the general improvement of health; in particular, the cultivar growing in Tongxiang City has the highest medicinal quality [29, 30]. These secondary metabolites are usually produced and accumulated under stressful environments, such as in Tongxiang City, which is a southeastern coastal city, in which the average salinity levels range from 0.1% to 0.4% and there is a huge area of coastal shoal resources under exploitation [31–33]. It has been reported that this cultivar of Hangbaiju plants has a moderate salt tolerance [27]; however, there has been no literature addressing how much benefit can be gained by the plants when they are inoculated with AMF under saline conditions. The purpose of this study was to answer the following questions: (1) Can inoculation of the plants with AMF enhance growth and nutrient uptake under saline conditions? (2) Which fungal species is the better option for improving salt tolerance?

## Materials and methods

### Experimental design

The experiment was designed with two factors: (1) four levels of mycorrhizal treatments, namely, control (non-mycorrhiza, NM), inoculation with *Funneliformis mosseae* (T. H. Nicolson & Gerd.) C. Walker and A. Schüßler (Fm), inoculation with *Diversispora versiformis* (P. Karst.) Oehl, G. A. Silva and Sieverd (Dv), and inoculation with combined inoculums of Fm and Dv (Fm+Dv); (2) three salinity levels, treated with 0, 50 and 200 mM NaCl treatments, which are equal to contents of 0, 0.1% and 0.4% NaCl. Thus, there were 12 treatment combinations arranged in a randomized complete block design with 5 replicates.

The experiment was conducted in a greenhouse located in Hangzhou, Zhejiang province in southeastern China (30°14′ N, 119°42′ E). Before the experiment, the inoculums of Fm and Dv were multiplied in an open-pot with a fine sand substrate. *Sorghum vulgare* L. was used as a host plant and was cultured for 5 months in the greenhouse [20]. Inoculums of Fm (BGC HUN03B) and Dv (BGC GD01C) were obtained from the Glomales Germplasm Bank in China (Institute of Plant Nutrient and Resources, Beijing Municipal Academy of Agriculture and Forestry Science). From late February to April 22, 2016, *Chrysanthemum morifolium* seedlings imported from Tongxiang City, Zhejiang Province, China, were cultivated in sterilized soils with γ-irradiation in does of 25 kGy [34]. On April 23, 2016, the experimental treatment began, and 60 similar-sized *C*. *morifolium* seedlings were transplanted into plastic pots (18 cm ×11 cm ×10 cm) each containing 1 kg of γ-irradiated medium (25 kGy) [34]. The potting medium consisted of peat and a local soil at a ratio of 1:2 (v/v). The medium had the following properties: 21.94 mg g^-1^ organic matter, 0.80 mg·g^-1^ total N, 0.32 mg·g^-1^ Olson P, pH 5.68 (water:soil = 5:1).

At the time of transplantation, 100 g of inoculum (Fm or Dv or the combination of Fm and Dv with each fungal species in 50 g of inoculum) was placed just below the roots of seedlings. The inoculum consisted of sand, spores and mycelium of Fm and/or Dv and infected root fragments [18] and contained an average of 80 AM fungal propagules per 10 g soil for both fungi. Control treatments received no AM fungal inoculums but received 100 ml of 100 g combined inoculum filtrate that was sieved through a 25 μm filter to provide similar microbial populations (excluding AMF) in all treatments. During the first 6 weeks of the treatment, the plants were grown without the addition of NaCl to obtain plants with functional mycorrhizas and avoid effects of salt on AMF establishment [18]. To avoid osmotic shock, NaCl was introduced gradually by successively adding 49 ml of the prescribed NaCl solution to each pot for 7 days; then, a total volume of 343 ml of the corresponding saline solution was added per pot in this experiment [20]. When leaching occurred, the leachate was collected and added to the soil to maintain salinity treatments near the target levels [35].

During the experiment, plants were grown under conditions with the mean temperature and relative humidity at 27.5°C and 65%, respectively (Thermo Datalogger, Campbell), and with a 14/10 h light/dark period along with 5 h of supplementary light per day, which was provided by 400 W high-pressure sodium lamps in the afternoon and evening [18]. Plants were watered individually with deionized water when needed and were supplied weekly with 10 ml of a nutrient solution based on the Hoagland solution [36] but with half of the normal concentrations of N and P [37]. The experiment was terminated on October 23, 2016. Shoots and roots were harvested separately.

### Measurements

At harvest, the plants were rinsed three times with deionized water and then separated into shoots and roots to determine the initial biomass. Leaf images were obtained with a scanner (Epson V330, Japan), and the leaf area was then measured with ImageJ (1.44p; National Institutes of Health, Bethesda, MD, USA). Subsequently, all plant shoots were dried at 70°C for 48 h for measurement of the dry masses. The N concentrations were determined with the elemental analyzer TruSpec CN (Leco, St. Joseph, MI, USA) according to the Austrian ÖNORM L 1080 protocol; the P concentrations were measured by the ammonium molybdate blue method [38].

Mycorrhizal colonization was detected by the following procedures: Roots from three randomly selected plants in each treatment were collected by gently washing out the soil under running tap water and rinsing three times in deionized water. A subsample of 0.5 g of fresh fine roots per plant was collected and cut into 1cm pieces [18]. Then, the root segments were stained using a modification of the Phillips and Hayman method [39]. Stained roots were analyzed under a dissecting microscope for the total mycorrhizal colonization percentage, arbuscule colonization percentage, and vesicle numbers per unit root length with the method described by Biermann and Linderman [40]. The remaining roots were washed clean of soil and dried at 70°C for 48 h. N and P concentrations were measured as described above for the shoots.

At the end of the experiment, the mycorrhizal dependency (MD) was calculated as follows [24]:

$$MD=\frac{DW_{AMF}-\bar{DW_{non-AMF}}}{DW_{AMF}}\times 100$$

Where DW_AMF_ is the dry weight of AMF plants; $\bar{DW_{non-AMF}}$is the mean value of dry weight of non-AMF plants.

### Statistical analysis

Two-way ANOVA (general linear model, SPSS 18.0, SPSS Inc., Chicago, IL, USA) was used to test the differences in the response parameters, with salt and AMF as fixed factors. Before the analysis, the data on root length, total dry weight, shoot N concentration, shoot P concentration, root N concentration, N:P ratio of shoot biomass, and the numbers of vesicles were square root-, log 10-, log 10-, log 10-, square root-, log 10-, and ln-transformed, respectively, based on Levene’s test for the equality of variance and the Shapiro-Wilk test for normality; the other data were analyzed without transformation. Additionally, the LSD multiple range test was used to compare the differences in the response variables between the treatments at *P* < 0.05.

## Results

### Mycorrhizal colonization

In this experiment, none of the Hangbaiju plants from the non-inoculated treatments were colonized by AMF. Plants inoculated with FM, Dv and the combined inoculums had total colonization percentages of 27.7%-56.9%, 34.6%-73% and 32.5%-60%, respectively. In addition, the extent of mycorrhizal colonization was significantly affected by salt and by the interaction effects of salt and AMF (Table 1). Except for the non-saline condition, there were no significant differences in the percentages of total mycorrhizal colonization and arbuscule colonization for the Hangbaiju plants inoculated with different fungal species (Table 1).

**Table 1:** Colonization of *Chrysanthemum morifolium* by arbuscular mycorrhizal fungi (AMF) under salt stress.

Treatments	Total colonization (%)		Arbuscule (%)		Numbers of vesicles (no./mm root length)		
0 mM NaCl+Fm	35.8 ± 9.2 cd		5.3 ± 1.2 ab		1.9 ± 1.0 bc		
0 mM NaCl+Dv	73.0 ± 5.2 a		12.0 ± 4.9 a		3.1 ± 1.4 ab		
0 mM NaCl+Fm+Dv	60.0 ± 9.6 ab		5.0 ± 2.1 ab		1.6 ± 0.5 bc		
50 mM NaCl+Fm	56.9 ± 7.0 abc		6.7 ± 3.2 ab		1.1 ± 0.4 bc		
50 mM NaCl+Dv	45.0 ± 9.2 bcd		2.2 ± 1.5 b		5.3 ± 0.9 a		
50 mM NaCl+Fm+Dv	47.4 ± 8.5 bcd		4.8 ± 2.7 ab		1.7 ± 0.2 bc		
200 mM NaCl+Fm	27.7 ± 4.0 d		3.6 ± 1.2 b		1.5 ± 0.8 bc		
200 mM NaCl+Dv	34.6 ± 4.6 cd		7.9 ± 1.5 ab		2.8 ± 1.8 abc		
200 mM NaCl+Fm+Dv	32.5 ± 7.5 d		1.0 ± 0.9 b		0 c		
Levels of significance							
	*F*	Sig	*F*	Sig	*F*	Sig	*df*
Salt	3.956	*	0.534	ns	0.068	ns	2
AMF	1.927	Ns	0.569	ns	1.773	ns	2
Salt × AMF	3.904	*	1.660	ns	2.539	ns	4
Error	14						

Data in the table are expressed as the mean ± SE. Values in columns followed by the same letter do not differ significantly at *P* <0.05 by LSD multiple range test. Fm, Dv and Fm+Dv represent inoculation with *Funneliformis mosseae*, *Diversispora versiformis* and the combination of *F*. *mosseae* and *D*. *versiformis*, respectively; *ns*, not significant at *P* > 0.05;

* P < 0.05.

### Plant growth

**Fig 1:**
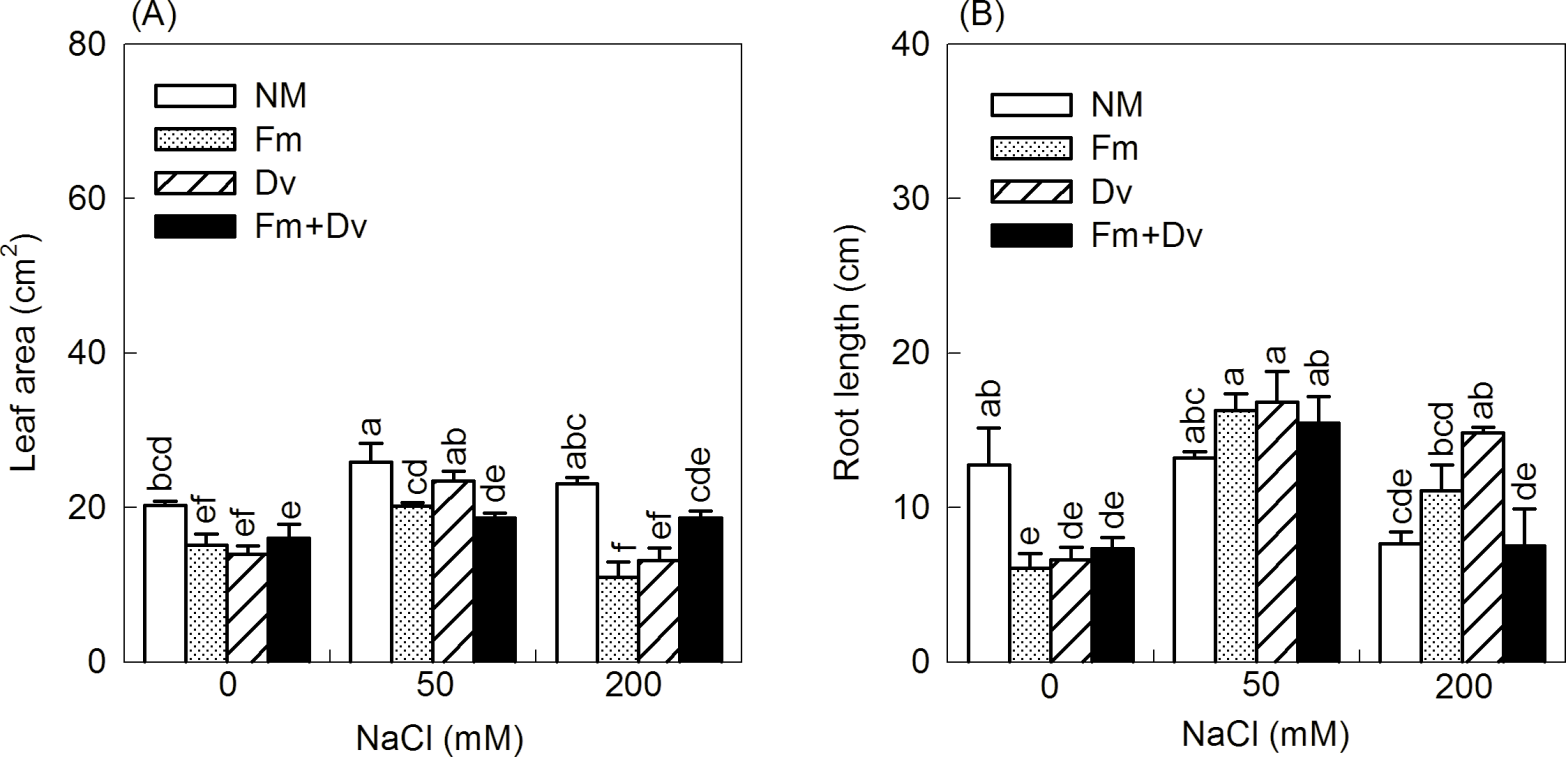
Effects of arbuscular mycorrhizal fungi on leaf area (A) and root length (B) of *Chrysanthemum morifolium* plants under 0, 50 and 200 mM NaCl. NM, Fm, Dv and Fm+Dv represent inoculation with no mycorrhizal fungi, *Funneliformis mosseae*, *Diversispora versiformis* and the combined inoculums, respectively. Values are presented as the mean ± SE. Values followed by the same letter do not differ significantly at P <0.05 by the LSD multiple range test.

Except for the root/shoot ratio, the growth parameters of the plants were significantly affected by salt; except for the root length and root dry weight, the growth parameters were significantly affected by AMF. In addition, the interaction of salt and AMF had significant effects on root length, root dry weight and total dry weight (Table 2). Compared to the non-colonized plants, inoculation with fungal species had no positive effects on the leaf area under saline or non-saline conditions (Fig 1A). In the absence of a saline treatment, inoculation with fungal species significantly decreased root length compared to the non-colonized plants, while under saline conditions, inoculation with fungal species had positive effects on root length, particularly at very high salinity levels (200 mM NaCl), and inoculation with Fm and Dv significantly increased root length, by 44% and 93%, respectively. However, the combined inoculums did not induce these effects (Fig 1B). In addition, among the non-colonized plants, the root length was 40% lower at 200 mM NaCl than in the non-saline treated plants, whereas among the AMF-colonized plants, the negative effect of NaCl was approximately 13% smaller with Fm and 16% larger with Dv (Fig 1B).

**Table 2:** Effects of salt, arbuscular mycorrhizal fungi (AMF) and their interactions on growth and nutrient parameters of *Chrysanthemum morifolium* plants.

Parameter	Salt	AMF	Salt × AMF
Leaf area	7.203** (2,30)	6.016** (3,30)	1.069^ns^ (6,30)
Root length	15.810*** (2,30)	0.512^ns^ (3,30)	4.703** (6,30)
Shoot dry weight	8.101** (2,30)	4.821** (3,30)	1.671^ns^ (6,30)
Root dry weight	8.044** (2,30)	1.578^ns^ (3,30)	2.698* (6,30)
Total dry weight	16.787*** (2,30)	2.931* (3,30)	3.812** (6,30)
Root/shoot ratio	0.745^ns^ (2,30)	4.810** (3,30)	0.977^ns^ (6,30)
MD	7.177** (2,35)	0.335^ns^ (2,35)	0.429^ns^ (4,35)
Shoot N concentration	3333.173*** (2,24)	129.896*** (3,24)	197.711*** (6,24)
Shoot P concentration	647.177*** (2,24)	490.471*** (3,24)	181.258***(6,24)
Root N concentration	394.410*** (2,24)	333.397*** (3,24)	534.881*** (6,24)
Root P concentration	808.550*** (2,24)	261.131*** (3,24)	26.549*** (6,24)
N:P ratio of shoot biomass	1574.122*** (2,24)	326.351*** (3,24)	170.134*** (6,24)

Data in the table are expressed as *F*-values and followed by *df* values in parentheses. MD: mycorrhizal dependence.

* *P* ≤ 0.05;

** *P* < 0.01;

*** *P* < 0.001;

ns, *P* > 0.05.

**Fig 2:**
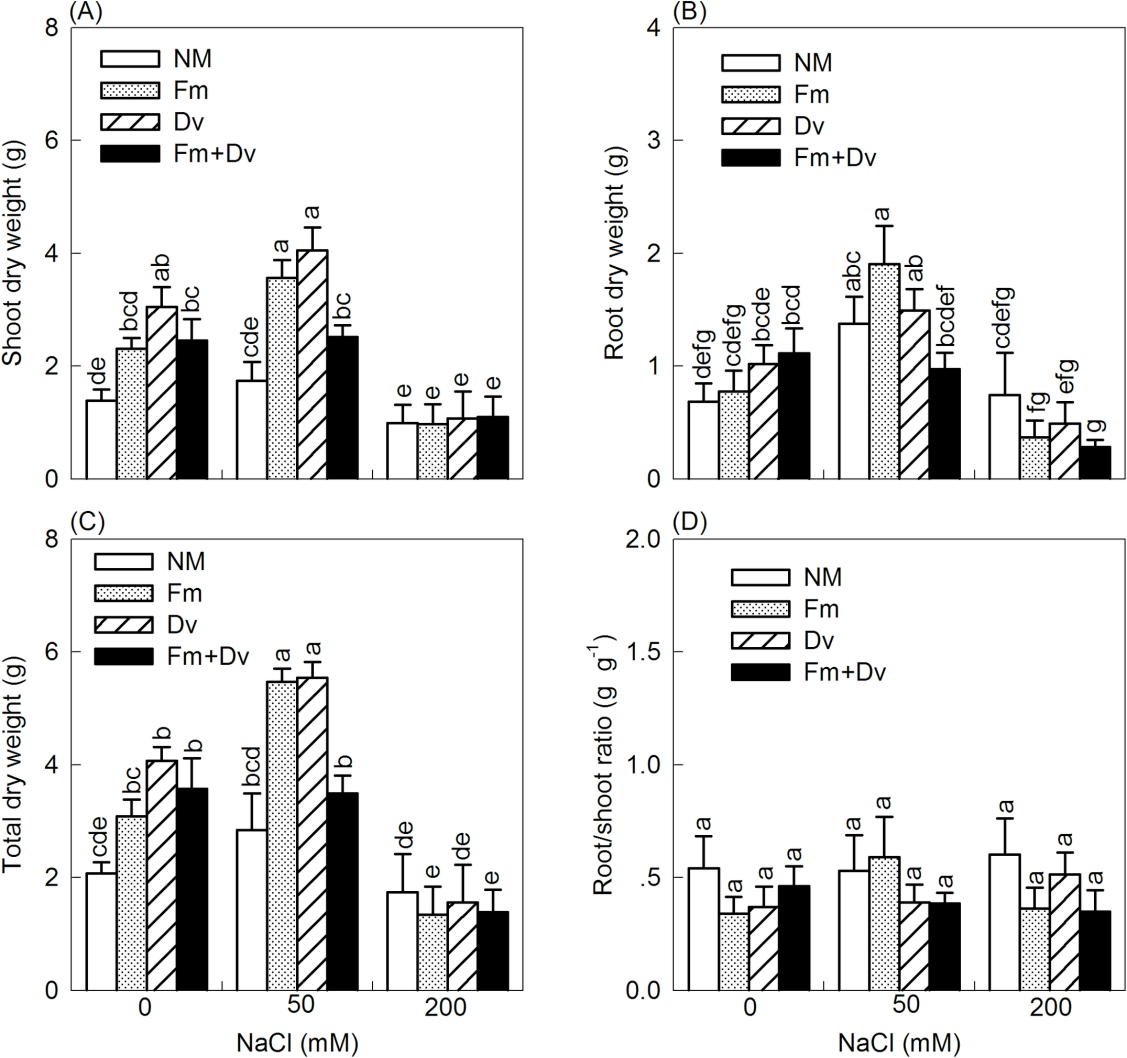
Effects of arbuscular mycorrhizal fungi on shoot dry weight (A), root dry weight (B), total dry weight (C) and root/shoot ratio (D) of *C*. *morifolium* plants under 0, 50 and 200 mM NaCl. Symbols are the same as in Fig 1.

Root colonization by fungal species significantly enhanced biomass development, especially under moderate saline conditions (50 mM NaCl) (Fig 2). Under non-saline conditions, compared to the non-inoculated plants, the shoot dry weight of the plants inoculated with Fm, Dv and the combined inoculums increased by 67%, 121% and 77%, respectively. In the presence of 50 mM NaCl, inoculations with Fm, Dv and the combined inoculums increased shoot dry weight by 105%, 133% and 45%, respectively, relative to the controls. In the absence of NaCl, Fm, Dv and the combined inoculums increased root dry weight by 13%, 49% and 63%, respectively, compared to the controls. In the presence of 50 mM NaCl, inoculations with Fm and Dv increased the root dry weight by 38% and 9%, respectively, relative to the controls, but the combined inoculums had no effect on the root dry weight. Clearly, under lower salt stress, the growth response to AMF was more effective at improving shoot development than root development. Under non-saline conditions, inoculations with Fm, Dv and the combined inoculums increased the total dry weight by 49%, 97% and 73%, respectively, compared to the non-inoculated plants. In the treatments with 50 mM NaCl, the total dry weights of the plants inoculated with Fm, Dv and the combined inoculums were higher than the controls by 92%, 95% and 23%, respectively. Nevertheless, at any given NaCl level, there were no significant differences in the root/shoot ratio irrespective of the colonizing fungi. Additionally, in the treatments with 200 mM NaCl, the shoot dry weight, root dry weight and total dry weight of inoculated or non-inoculated plants decreased more substantially than under non-saline conditions, indicating that inoculating plants with AMF had no positive effects on biomass development under severe salt stress.

**Fig 3:**
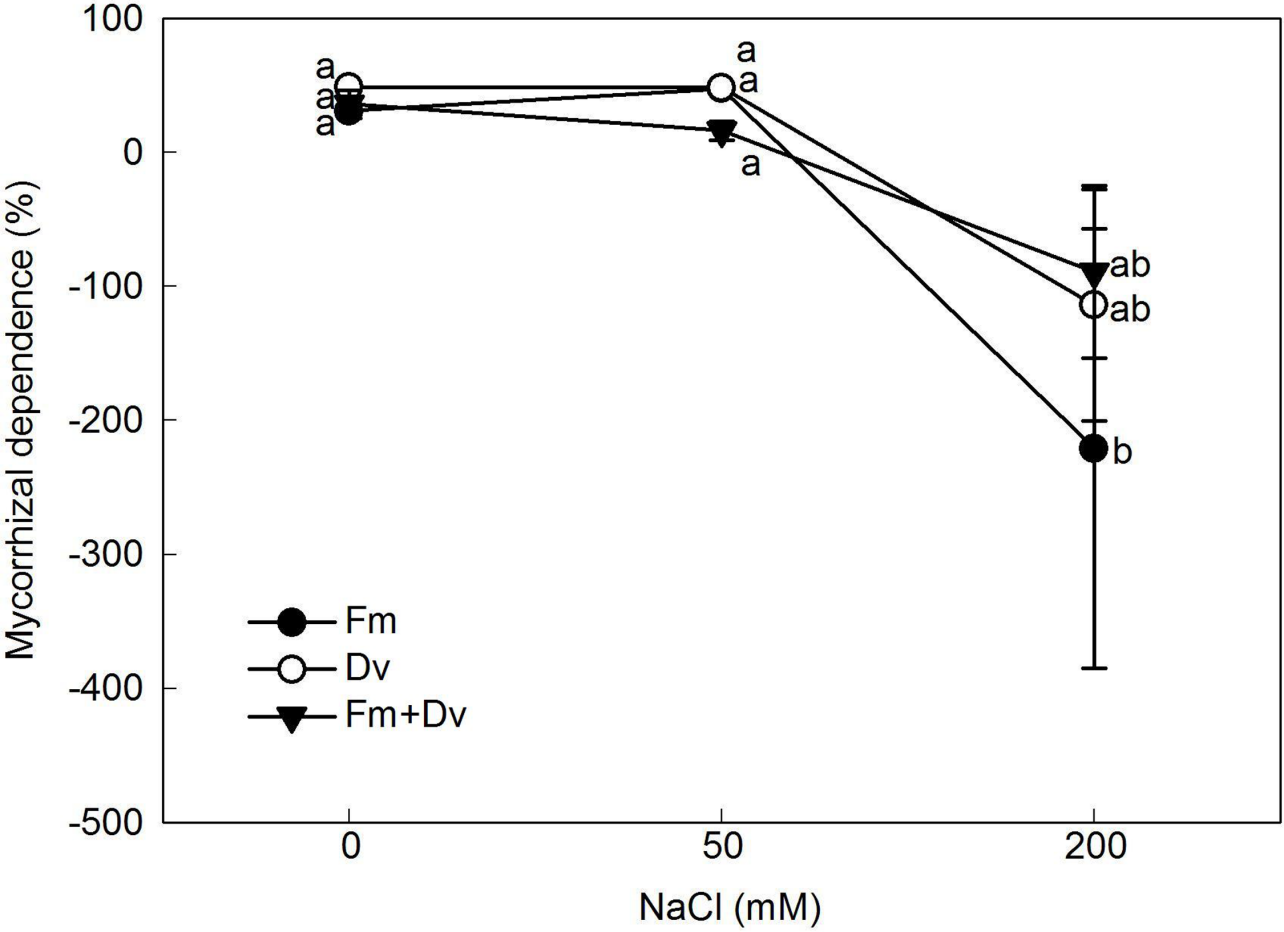
The mycorrhizal dependence of *C*. *morifolium* plants under 0, 50 and 200 mM NaCl. Symbols are the same as in Fig 1.

Furthermore, with the increase in salinity, the percentage of mycorrhizal dependence (MD) stably persisted under conditions of no or moderate salinity (0 or 50 mM NaCl) but greatly decreased under severe salt stress (200 mM NaCl) (Fig 3). In addition, the dependence of plant growth on mycorrhizal symbiosis was greatest when plants were colonized by Dv.

### Plant nutrients

**Fig 4:**
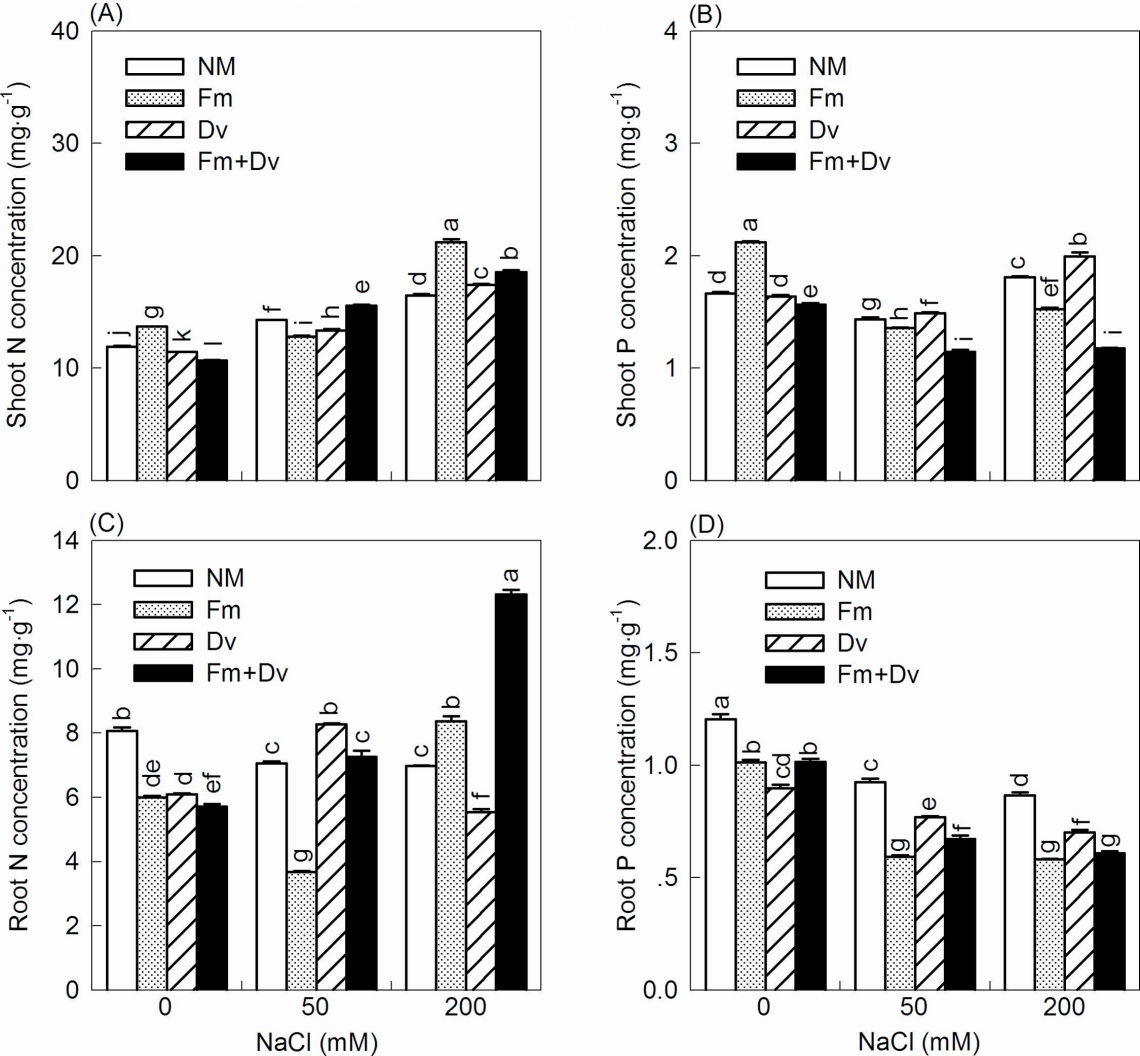
Effects of arbuscular mycorrhizal fungi on shoot N concentration (A), shoot P concentration (B), root N concentration (C) and root P concentration (D) of *C*. *morifolium* plants under 0, 50 and 200 mM NaCl. Symbols are the same as in Fig 1.

Salt, AMF and the interaction of salt and AMF had significant effects on the N and P concentrations of tissues and on the N:P ratio in shoot biomass (Table 2). With increasing salinity, the shoot N significantly increased, the root P concentrations decreased, and the shoot P and root N concentrations varied greatly; nevertheless, the effects of AMF colonization on nutrient uptake varied greatly at each NaCl level (Fig 4). Compared to the controls, inoculation with Fm increased shoot N concentration by 15%, whereas inoculation with Dv and the combined inoculums decreased it by 4% and 10%, respectively, under non-saline conditions. In the treatments with 50 mM NaCl, the shoot N concentration decreased by 10% and 7% with Fm and Dv, respectively, while that of the plants with combined inoculums increased by 9% relative to non-inoculated plants. Compared to the non-inoculated plants, under 200 mM NaCl and with Fm, Dv and the combined inoculums, the shoot N concentration of inoculated plants increased by 29%, 6% and 13%, respectively. Under non-saline conditions, inoculation with Fm significantly increased the shoot P concentration by 27%, while inoculation with Dv and the combined inoculums had no positive effects on shoot P concentration compared to the non-inoculated plants. Under saline conditions, only inoculation with Dv had positive effects on shoot P concentrations compared to non-inoculated plants. In addition, under non-saline conditions, root N concentrations were not improved compared to the controls by inoculation with any of the fungal species. In the treatments with 50 mM NaCl, inoculation with Fm decreased the root N concentration by 48%, whereas inoculations with Dv and the combined inoculums increased it by 17% and 13%, respectively, relative to the non-inoculated plants. Nonetheless, in the treatments with 200 mM NaCl, inoculation with Fm and the combined inoculums increased the root N concentration by 20% and 77%, respectively, whereas inoculations with Dv decreased it by 20% relative to the non-inoculated plants. At any given NaCl level, inoculation with any of the fungal species significantly decreased the root P concentration compared to the controls.

**Fig 5:**
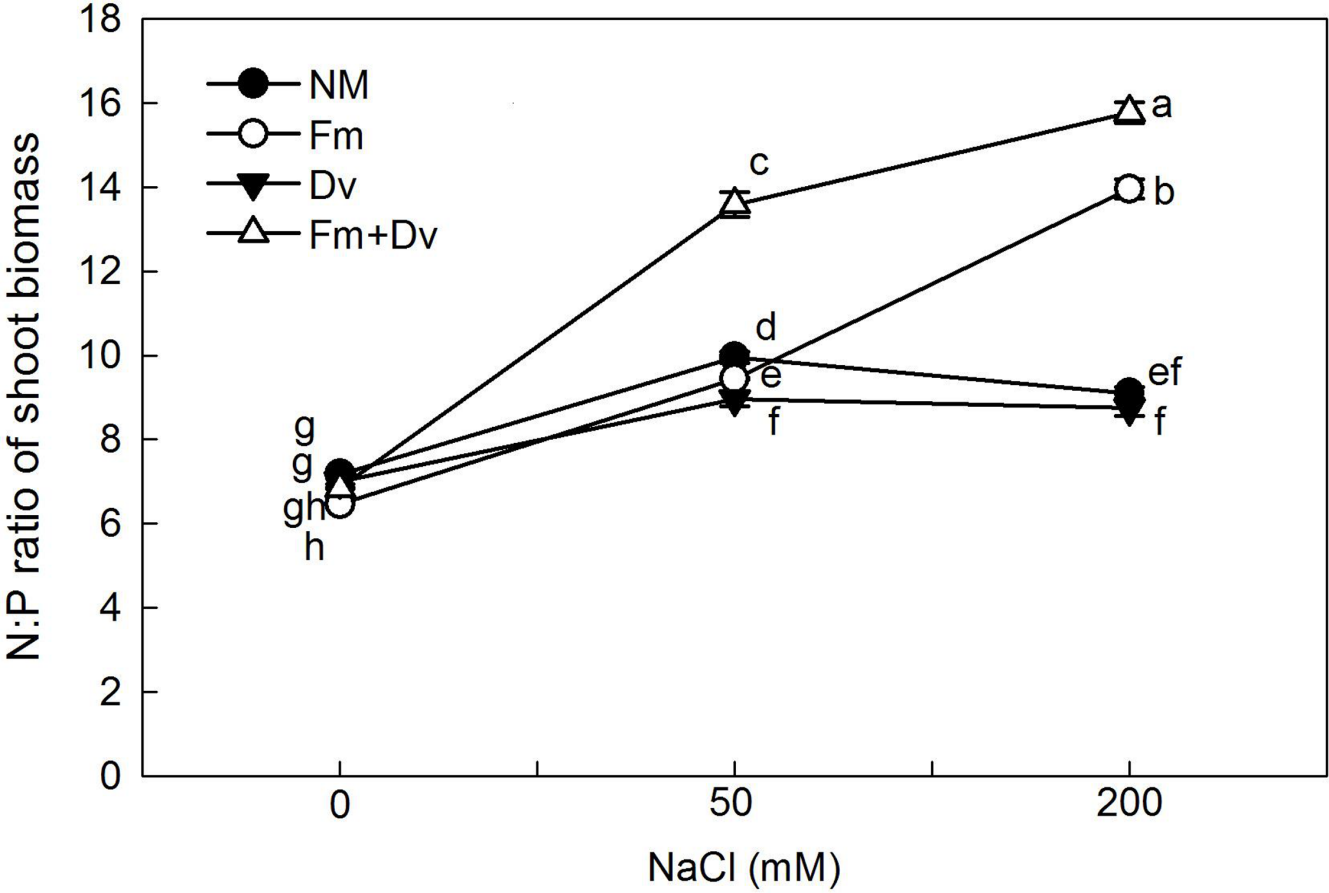
Effects of arbuscular mycorrhizal fungi on the N:P ratio of *Chrysanthemum morifolium* plants under 0, 50 and 200 mM NaCl. Symbols are the same as in Fig 1.

In addition, with increasing salinity, the shoot biomass N:P ratios of plants inoculated with Fm and the combined inoculums significantly increased, from 7 to 16, while the shoot biomass N:P ratios of non-inoculated plants and Dv plants first increased and then decreased, with values remaining under 10 (Fig 5).

## Discussion

In this study, inoculation by fungal species, especially by Dv, induced plants with longer root length, higher shoot and root dry weight, higher total dry weight, and higher root N concentration under moderate NaCl conditions compared with non-inoculated plants, indicating that AMF can somehow enhance the growth and root N uptake under moderate saline conditions. Additionally, mycorrhizal dependence (MD) maintained positive values under moderate salinity. These results showed that *C*. *morifolium* plants were highly dependent on AMF colonization to reach optimal growth under moderate saline conditions, and the most active fungus was Dv. In addition, as Feng et al. [18] noted, the positive effects of AMF on plants were not salinity stress dependent, as beneficial effects occurred irrespective of the presence of salt stress.

There were significant improvements in the dry matter development of the plants under moderate salinity (50 mM NaCl), which are consistent with results for the tomato cultivar Piazar [19] and for sugar beet [41]. Hajiboland et al. suggested that these beneficial effects are attributed to the replacement of K by Na in plants [19]. However, dry matter production was significantly reduced under severe salt stress (200 mM NaCl). Plants growing in saline soils are subject to three primary physiological stresses [4,7]: First, the toxic effects of specific ions such as sodium and chloride disrupt normal physiological processes, such as photosynthesis, respiration, and protein synthesis; Second, there is a risk of “physiological drought” caused by osmotic stress, which reduces the water available to the plant; Third, nutrient imbalance is induced by high uptake of Na and Cl in plants. In this experiment, the effects of salinity on growth could be enhanced by mycorrhizal colonization (Figs 1 and 2). These findings are consistent with previous reports for AMF-colonized plants under saline conditions [11,21,42–45]. Longer root length and more biomass accumulation of AMF-inoculated plants under mild salinity are adaptive strategies for acquiring water and nutrients [7]. It has been suggested that improving plant P uptake is the most important salinity stress tolerance mechanism in AMF-inoculated plants [5,11,22,46]. However, some studies have shown that mycorrhizal plants grow better than non-mycorrhizal plants under salt stress even when the P status is similar between the mycorrhizal and non-mycorrhizal plants [23,47]. In addition, Ruiz-Lozano et al. [47] argued that the mechanism underlying AMF plant growth improvements under saline conditions is based on physiological processes (such as photosynthesis, transpiration, and water use efficiency) rather than on nutrient uptake (N or P). Additionally, Feng et al. [18] suggested that it is the higher soluble sugar accumulation in mycorrhizal plants relative to non-mycorrhizal plants and not the P status that enhances salt resistance.

Our study shows that the enhancement of nutrient uptake with AMF inoculation mainly appeared with the N concentration, not the P concentration (Fig 4). It has been reported that the application of AMF may improve nitrogen assimilation by host plants [10]. For example, Giri and Mukerji [48] recorded a higher accumulation of N in the shoots of mycorrhizal *Sesbania grandiflora* and *S*. *aegyptiaca* than in non-mycorrhizal control plants. The increased nutrient uptake observed may be explained by the fact that the hyphae of AMF often penetrate 7 cm or more into the soil beyond the rhizosphere, where they can absorb water and nutrients under different osmotic potentials than those at the root surface [11,49]. Improved N uptake may help to reduce the toxic effects of Na^+^ ions by regulating its uptake and by indirectly helping to maintain the chlorophyll content of the plant [9,10]. Additionally, the biomass N:P ratio in this experiment provides additional support for the enhancement of N uptake as the underlying mechanism by which AMF reduce saline stress in plants. The biomass N:P ratio has been mainly used to assess whether N or P is more limiting for biomass production, and usually, N:P ratios < 10 and > 20 correspond to N- and P-limited biomass production [50]. As salinity increased, the biomass N:P ratios of non-inoculated plants and Dv plants stayed under 10, indicating that they probably undergo N-limited biomass production. For plants inoculated with Fm and the combined inoculums, the N:P ratios increased from 7 to 16, indicating that biomass production is still N-limited, and there is a transition between N- and P-limited biomass production.

Furthermore, AMF differed in their ability to enhance growth and nutrient uptake even though there were small differences among the fungal species in terms of their ability to colonize the roots (Table 1; Fig 4). This finding is in agreement with previous studies [24,49]. Specific mechanisms that result in functional differences between AMF could be expected because of differences in fungal characteristics such as length of external mycelia, distribution of hyphae, and/or nutrient translocation [24]. In fact, the positive effects of AMF on the growth and nutrient uptake of *C*. *morifolium* plants under lower saline conditions may be inhibited under severe salt stress because soil salinity can also influence the growth and activity of AMF [4]. With increasing NaCl levels, the mycorrhizal colonization varied from the highest percentage at 73% with Dv under no added NaCl condition to the lowest approximately 28% with Fm under 200 mM NaCl (Table 1). The reduced colonization with salt application is consistent with the observations of other studies [22,35,51]. Moreover, the mycorrhizal dependency decreased with increasing salinity, especially under severe salt stress (200 mM NaCl, suggesting that the establishment of the association between mycorrhizal fungi and the plants seemed to provide benefits for plants only under moderate salt stress and not under conditions of high salinity (Fig 3). The results are somewhat consistent with mycorrhizal *Cajanus cajan* (L.) under the similar moderate salinity levels as 4 and 6 dS m^-1^ ECe [9].

The above results show that AMF differ in their ability to enhance growth and nutrient uptake of *C*. *morifolium* plants under saline conditions, but the Dv fungal inoculation was more active and effective than the other inoculum treatments. There are specific compatibility relationships that exist between symbionts, underscoring the importance of host-endophyte selection to maximize the growth and nutrition of plants. AMF symbiotic efficiency attributed to plants is dependent on the plant species and the AMF species, and the selection of the most suitable AMF for a specific plant is of practical importance for improving AMF effectiveness under specific environmental conditions [24].

## Conclusions

This study determined that colonization with fungal species improved the growth and root N uptake of *C*. *morifolium* plants under moderate salinity conditions. Nevertheless, AMF differ in their effects on *C*. *morifolium* plants under saline conditions, and Dv was the most active fungus. These observations consolidate evidence for the potential use of Dv to enhance *C*. *morifolium* plant growth under moderate salt stress and may pave the way to take advantage of this symbiotic association in biotechnological practice.
